# Hot Water Vaginal Injections

**Published:** 1880-09

**Authors:** C. A. Kirkley

**Affiliations:** Toledo, O.


### HOT WATER VAGINAL INJECTIONS.

C. A. KIRKLEY, M.D. TOLEDO, O.

The primary effect of cold upon living tissue is to produce capillary congestion, reflex action is excited, the blood vessels contract, and the blood within the part is diminished for the time being. When reaction takes place, however, relaxation of the parts ensues, there is more conjestion than before, with engorgement of both arteries and veins.

The organs of generation are abundantly supplied with blood vessels, nerves, and absorbents. The arterial supply is from the spermatic, given off from the aorta below the renal, and the uterine, given off from the internal iliacs, their branches freely anastomosing. The veins follow the course of their respective arteries. The nervous supply is derived from the sacral plexus of the cerebro-spinal system, and from the great sympathetic system. The absorbents run into the sacral and lumbar glands of the groin. The minutest capillaries are covered by nerve filaments. In disease of those organs so liberally supplied from such important sources, great functional disturbance, as well as impairment of nutrition must result. The connective tissue of the pelvis must necessarily be affected by any disturbance of the circulation, for in no other part of the body is there such a net-work of vessels within the same space. The tissues are all erectile, and in prolonged disease the veins especially become greatly distended, even viricose, from obstruction to the circulation. In hypertrophic conditions of the uterus, the enlargement and malnutrition are due rather to obstruction to the return of venous blood, than the increased supply of arterial. The veins therefore lose their tone, and there is almost a stasis of blood. The weight of the organs and the secretion from them is increased. Such a condition implies impairment of nutrition and loss of tone in the diseased parts, and recovery is impossible unless they are restored. To this end constitutional means are necessary, but they will accomplish little without the aid of those local means which excite direct and reflex action within the vessels and nerves, producing their contraction, thereby increasing their tonicity and improving the nutrition of the parts.

Heat applied for a short time is not so prompt an agent to produce this necessary contraction as cold, but its continuous application for a length cf time does so more thoroughly and permanently. Its primary effect is to produce relaxation and congestion of the parts, but its permanent effect is the reaction from heat when prolonged in its application. Capillary action is increased, the veins are stimulated, the larger vessels are toned, their calibre diminished, and congestion proportionately relieved. It was formerly believed that relaxation and congestion followed the application of heat, and such is its immediate effect, but when prolonged it has an opposite effect, viz: tonic. Hot poultices are not applied to increase congestion, but to diminish it by exciting the blood vessels to contract. That this is true is proven by the shriveled condition of the part when the poultice is removed. The washerwoman's hands are enlarged when first put into hot water from a determination of blood to them, but as is well known to every one, they become shrivelled when kept in the water for a length of time.

If the hands are placed in cold water the skin will shrink from contraction of the blood vessels; but when reaction occurs, the parts contain a larger quantity of blood than was driven out, congestion is present, though only slight in degree. Hence the immediate effect of cold is contraction, and when reaction occurs there is dilitation; the reverse however is true where heat is applied, dilitation first occurs, then contraction.

To Emmet is due the credit of having first used hot water as a therapeutic agent in a systematic manner, and the views as to its action expressed in this paper, are mainly derived from his work, and are entirely in accord with the experience of those who have given the agent a thorough trial. In my own experience no one agent has been more efficient in the treatment of all forms of uterine enlargement, inflammation of the cervix, vaginitis, pelvic cellulitis, cystitis, etc., and it has been beneficial in proportion as the details in its application have been attended to.

The temperature of the water should be that which is most agreeable to the patient. It need not be more than tepid in the beginning, and gradually increased in some cases to 100, 110, 115, and even 120 degrees. It is remarkable what tolerance its use will soon establish, and frequently the higher temperature, so that the parts are not actually scalded, the greater the comfort and relief. Should a metallic syringe nozzle be used, more discomfort will be experienced from it than from the high temperature of the water; a nozzle of some nonconducting material therefore is preferable. Patients have remarked that they could not hold their hands in the water used.

An ordinary Davidson or Mattson syringe is a suitable instrument for giving an injection, and when not at hand a substitute can be improvised by putting a spout two or three inches long at or near the bottom of a tin vessel, two or three gallons capacity; the spout should be inclined downwards, and to its end may be attached a rubber hose of any length desired. When in use the vessel may be hung upon a hook or nail in the wall, or placed upon a chair set upon a table. This contrivance is preferable to the syringe, when a douche is required. I first saw this device used by Dr. Collamore, of this city, to wash out the urinary bladder of the male. A male catheter, however, was attached to the hose, instead of the nozzle necessary for vaginal injections, for which purpose I have frequently used it, and can testify as to its convenience and efficiency.

Dr. Fowler, of Youngstown, O., a consistent advocate of hot water applications, recommends what he calls the fountain douche. He says : "A twelve-quart wooden bucket, five feet of rubber tubing, half an inch calibre, an old-fashioned cot-bed, a tub and two glass tubes—mostly available in any household, or easily obtained, are all we require to construct an efficient fountain douche." Instead of the metalic nozzle of the common syringe, he uses a glass tube, six inches long, both ends melted, and for the spout one two inches long to attach the rubber tube to the side of the bucket, half an inch from the bottom. While in use, the bucket is elevated to a chair upon an ordinary table. The flow of water is regulated by a clamp. The bed has an opening through the canvas and a tunneled rubber cloth under the nates to convey the water from the vulva into a receiver under the cot.

The most convenient position for the patient is upon her back across the bed, the nates close to the edge, each foot upon a chair, and the hips elevated by a folded quilt or comforter, covered with oil silk or rubber tissue, folded in such a way as to convey the water into a tub or bucket, previously placed under her. Should there be any objection to changing the patient in bed, the hips may be elevated with a bed-pan, which will also receive the water. A vessel large enough to hold two or three gallons may be placed conveniently near her, and a Davidson, Mattson or other syringe used. Two gallons or more may be injected at one time. With the hips elevated in this manner, the patient has the benefit of gravity, in relieving the engorged pelvic vessels, at least for the time being.

A patient can not well give herself an injection, and it should usually be given at night on going to bed. Emmet suggests the knee-chest position as the best to get the full benefit of hot water, as in addition to gravity we have atmospheric pressure to a d in relieving the engorged veins, and the vagani being distended and the folds entirely obliterated, it is more thoroughly applied. The patient and bedding may be protected by oil cloth folded in such a manner as to convey the water off, and placed close against the pubes. Patients usually denominate this position "standing on the head," and some rather object at first, but the relief is so great that any objection is very soon forgotten.

As far as I am aware, hot water vaginal injections have not been used as their value demands, and if this paper should encourage members of the society to investigate the subject and give it a fair trial, its object will have been attained. It is applicable under almost all circumstances, when inflammation exists, and has been of more value in my hands than all other local means combined. To be beneficial, however, it must be properly applied.—*Toledo Medical and Surgical Journal.*

